# Electroconvulsive shock attenuated microgliosis and astrogliosis in the hippocampus and ameliorated schizophrenia-like behavior of Gunn rat

**DOI:** 10.1186/s12974-016-0688-2

**Published:** 2016-09-02

**Authors:** Erlyn Limoa, Sadayuki Hashioka, Tsuyoshi Miyaoka, Keiko Tsuchie, Ryosuke Arauchi, Ilhamuddin A. Azis, Rei Wake, Maiko Hayashida, Tomoko Araki, Motohide Furuya, Kristian Liaury, Andi J. Tanra, Jun Horiguchi

**Affiliations:** 1 Department of Psychiatry, Shimane University Faculty of Medicine, 89-1 Enya-cho, Izumo, 693-8501 Japan; 2 Department of Psychiatry, Hasanuddin University Faculty of Medicine, Jl. Perintis Kemerdekaan Km. 10, Makassar, 90245 South Sulawesi Indonesia; 3 Department of Developmental Biology, Shimane University Faculty of Medicine, 89-1 Enya-cho, Izumo, 693-8501 Japan

**Keywords:** Electroconvulsive shock, Schizophrenia, Microglia, Astrocytes, Hippocampus, Gunn rat

## Abstract

**Background:**

Although electroconvulsive therapy (ECT) is regarded as one of the efficient treatments for intractable psychiatric disorders, the mechanism of therapeutic action remains unclear. Recently, many studies indicate that ECT affects the immune-related cells, such as microglia, astrocytes, and lymphocytes. Moreover, microglial activation and astrocytic activation have been implicated in the postmortem brains of schizophrenia patients. We previously demonstrated that Gunn rats showed schizophrenia-like behavior and microglial activation in their brains. The present study examined the effects of electroconvulsive shock (ECS), an animal counterpart of ECT, on schizophrenia-like behavior, microgliosis, and astrogliosis in the brain of Gunn rats.

**Methods:**

The rats were divided into four groups, i.e., Wistar sham, Wistar ECS, Gunn sham, and Gunn ECS. ECS groups received ECS once daily for six consecutive days. Subsequently, prepulse inhibition (PPI) test was performed, and immunohistochemistry analysis was carried out to determine the activation degree of microglia and astrocytes in the hippocampus by using anti-CD11b and anti-glial fibrillary acidic protein (GFAP) antibody, respectively.

**Results:**

We found PPI deficit in Gunn rats compared to Wistar rats, and it was significantly improved by ECS. Immunohistochemistry analysis revealed that immunoreactivity of CD11b and GFAP was significantly increased in Gunn rats compared to Wistar rats. ECS significantly attenuated the immunoreactivity of both CD11b and GFAP in Gunn rats.

**Conclusions:**

ECS ameliorated schizophrenia-like behavior of Gunn rats and attenuated microgliosis and astrogliosis in the hippocampus of Gunn rats. Accordingly, therapeutic effects of ECT may be exerted, at least in part, by inhibition of glial activation. These results may provide crucial information to elucidate the role of activated glia in the pathogenesis of schizophrenia and to determine whether future therapeutic interventions should attempt to up-regulate or down-regulate glial functions.

**Electronic supplementary material:**

The online version of this article (doi:10.1186/s12974-016-0688-2) contains supplementary material, which is available to authorized users.

## Background

Electroconvulsive therapy (ECT) has been used as a treatment for mental disorder since the 1930s [1] because of its effectiveness and the fast action in several psychiatric disorders such as bipolar disorder, major depression, and schizophrenia accompanied by catatonia, extreme depression, mania, and other affective components [2, 3]. Nevertheless, views on ECT vary; some researchers consider that it is probably ineffective and certainly causes brain damage, while others think it is completely safe and the most effective treatment available in psychiatry [4]. Both the generalized seizure and the dose of electricity used seem to be important for the therapeutic effect of ECT, which has multiple, varied, and lasting effects on the CNS [5, 6]. However, the exact mechanism of therapeutic action of ECT remains unknown.

Recent studies indicate that ECT affects the immune system. A single ECT has been reported to up-regulate the immune system, causing elevated levels of pro-inflammatory cytokines [7]. On the other hand, repeated ECT administrations appear to down-regulate the immune system as shown by a reduction of the plasma levels of tumor necrosis factor (TNF)-α [8]. Pro-inflammatory cytokines, including TNF-α, are released from immune-related glial cells in the CNS [9, 10].

A change in immune system functions associated with glial activation may be involved in the pathogenesis of schizophrenia [11, 12]. In fact, several postmortem brain studies and positron emission tomography studies on schizophrenic patients have indicated an increase in microglial activation [13–18]. In addition, activated astrocytes have been reported in the postmortem brains of schizophrenia [19, 20]. Therefore, it is tempting to determine the effect of ECT on the activation of microglia and astrocytes.

It is believed that there is a relationship between hyperbilirubinemia and schizophrenia. Schizophrenic patients have a significantly higher frequency of hyperbilirubinemia relative to patients with other psychiatric disorders and to the general healthy population [21, 22]. Gunn rats, a mutant of the Wistar strain, have a genetic deficiency in glucuronyl transferase. This deficiency leads to high levels of unconjugated bilirubin in their blood and various tissue, including the brain [23, 24]. Our previous studies have revealed that Gunn rats show a behavioral abnormality similar to schizophrenia with deficits in prepulse inhibition (PPI) [25, 26]. Furthermore, we have shown that microglia are activated in the hippocampal dentate gyrus (DG) of Gunn rats [27, 28]. Based on these findings, the present study evaluated the effects of electroconvulsive shock (ECS), an animal model of ECT, on schizophrenia-like behavior, as well as on microgliosis and astrogliosis in the hippocampus of Gunn rats.

## Methods

### Animals

Six-week-old male homozygous (j/j) Gunn rats and male Wistar rats (Japan SLC, Inc., Japan) were used in this study. The rats were housed under standard conditions with a room temperature (RT) of 23 ± 2 °C, humidity of 55 ± 5 %, and 12-h light/12-h dark cycle (light phase 7:00 to 19:00) and with free access to food and water. Two weeks before starting the experiment, the rats underwent a handling procedure once daily to reduce stress during the experiments. All procedures were performed with the approval of the Shimane University Animal Ethics Committee, under the guidelines of the National Health and Medical Research Council of Japan.

### ECS procedure

Animals were divided into four groups: Wistar sham group (WS), Wistar ECS group (WE), Gunn sham group (GS), and Gunn ECS group (GE) in order to get the brain samples from sham-treated groups and from ECS-treated groups. To avoid stress or pain induced by the ECS procedure, each rat was first anesthetized in a halothane inhalation chamber (4 % for initial induction) with an oxygen flow rate of 2–4 L/min [29]. After the rats fell asleep, the rats were taken out from the chamber and anesthesia was continued by putting a halothane inhalation mask (2 % for maintenance) with an oxygen flow rate of 2–4 L/min. In every ECS procedure, electric shock was given under anesthesia by halothane inhalation mask.

ECS was administered transcranially via bilateral ear clip electrodes using an E.C. Stimulator MK-810 (Muromachi Kikai Co., Ltd., Japan). The stimulus was a sine wave pulse, 100 v, 60 Hz, 50 mA, 1.5 s. Each stimulation resulted in a typical tonic-clonic seizure lasting for less than 10 s (for review, see Additional file 1). The ECS group received an ECS treatment once daily for 6 consecutive days. The sham-treated control groups were handled identically to the ECS groups including anesthesia except that no current was delivered.


**Additional file 1.** The ECS group received an ECS treatment once daily for 6 consecutive days and each stimulation resulted in a typical tonic-clonic seizure lasting for less than 10 seconds. (MP4 5677 kb)

### Hearing ability and PPI of startle response

A startle response measurement system (SR-Lab, San Diego Instruments, Inc., CA) was used as described previously [25, 28]. Each animal (*n* = 8 for each group) was placed in a plexiglass cylinder where it was exposed to white background noise at 65 dB for a 5-min acclimatization period. This was followed by four types of trials: (1) pulse (P) alone which consists of a 20-ms burst of white noise at 120 dB; (2) a 20-ms burst of white noise at 70 dB followed by a 20-ms white noise at 120 dB (70 PP + P); (3) a 20-ms burst of white noise at 80 dB followed by a 20-ms white noise at 120 dB (80 PP + P); and (4) background noise only (no stimulus). The interval between prepulse and pulse was set at 100 ms. Trials were given in a pseudo-random order with variable intervals (5–45 s) between trials. Startle response was measured in a session which consists of 50 trials. We identified the maximum amplitude of the startle response (Vmax) in each trial. The mean of Vmax was used to calculate the degree of PPI (%PPI). The percentage of PPI was defined as the magnitude of inhibition due to the startle amplitude that was induced by the prepulse. %PPI = [1 − (startle magnitude after prepulse − pulse pair/startle magnitude after pulse only)] × 100.

In order to determine whether Gunn rats have intact hearing, Wistar rats and Gunn rats without any treatment (*n* = 3 for each group) were also tested with the startle response measurement system. Each animal was exposed to white background noise at 65 dB for a 5-min acclimatization period. This was followed by five types of trials: (1) pulse alone which consists of a 20-ms burst of white noise at 85 dB; (2) pulse alone which consists of a 20-ms burst of white noise at 90 dB; (3) pulse alone which consists of a 20-ms burst of white noise at 100 dB; (4) pulse alone which consists of a 20-ms burst of white noise at 110 dB; and (5) background noise only (no stimulus). Trials were given in a pseudo-random order with variable intervals (5–45 s) between trials. Startle response was measured in a session which consists of 50 trials. We measured Vmax in each trial and compared the mean of Vmax between the Wistar rat group and the Gunn rat group.

### Immunohistochemistry

After the behavior tests, animals underwent deep intraperitoneal anesthesia with sodium pentobarbital (80 mg/kg body weight) and were perfused transcardially with 500 mL of physiological saline, followed by 500 mL of 10 % formalin. The brains (*n* = 6 for each group) were quickly removed and were fixed in a solution of 10 % formalin at RT for 4 h. The brains were immersed overnight in a cold solution of 20 % sucrose and then were cut into 40-μm-thick serial sections using a sliding microtome (Microm HM 430, Thermo Scientific, Germany). Fifteen to 18 slices from each rat were processed for each marker.

The free-floating brain sections were incubated in 1 % H_2_O_2_ for 30 min at RT and then were pre-incubated with 0.1 M phosphate buffer containing 3 % bovine serum albumin, 0.4 % Triton X, and 1.5 % goat serum for 1 h at RT. The sections were incubated for 3 days with the rabbit anti-glial fibrillary acidic protein (GFAP) antibody (1:2000, Abcam plc., Cambridge, UK) at 4 °C. Similar procedures were done with the mouse anti-CD11b antibody (1:500, Abcam plc., Cambridge, UK) except that 1.5 % horse serum was used instead of goat serum. Subsequently, the sections were incubated for 1 h with biotinylated anti-rabbit (or anti-mouse) IgG antibody (1:200, standard ABC kit, Vector lab, Inc., CA) at RT. For diaminobenzidine (DAB) staining, the sections were incubated for 1 h at RT in PBS containing the avidin-biotin peroxidase complex solution. The immunoreactivity in the sections was developed by incubating in PBS containing 0.5 % DAB and 0.1 % H_2_O_2_ for 10 min. The DAB reaction was halted by PBS. The sections were mounted onto gelatin-coated slides and were sunk in graded alcohol baths for dehydration.

For immunofluorescent staining, the sections were incubated with the rabbit anti-GFAP antibody (1:500) followed by incubation with Cy3 conjugated anti-rabbit IgG antibody (1:1000, Jackson ImmunoResearch, PA) and 4',6-Diamidino-2-Phenylindole (DAPI) (0.5 μg/mL, Sigma-Aldrich, MO) for 3 h at RT. The sections incubated with mouse anti-CD11b antibody (1:500) were incubated in Alexa Fluor 488 conjugated anti-mouse IgG antibody (1:500, Life Technologies Eugene, OR) and DAPI (0.5 μg/mL) for 3 h at RT.

### Image analysis

**Fig. 1:**
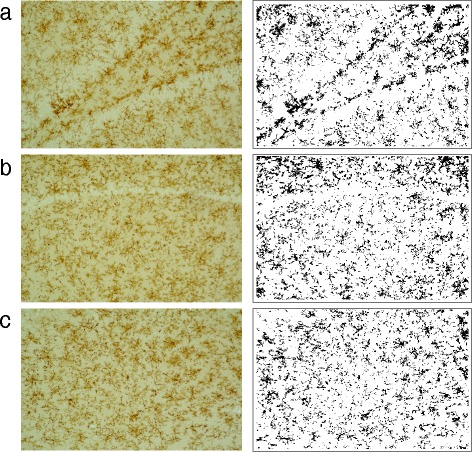
Representative images of DAB staining and of black-/white-pixel conversion in the DG area (**a**), the CA1 area (**b**), and the CA3 area (**c**)

The intensity of astroglial and microglial immunoreactivity in the DAB staining was measured by a computer-assisted image analysis program (Image J 1.47v, Wayne Rasband, National Institutes of Health, MD). GFAP- or CD11b-labeled glial cells were examined under a light microscope (Nikon, Eclipse Cί, Japan) with a 20× objective lens. Images were captured from three areas within the hippocampus, namely the dentate gyrus (DG), the cornu ammonis (CA)1, and the CA3. Twenty images were captured bilaterally with a digital Nikon 1 J1 camera from each area (10 images from the left hemisphere and 10 images from the right hemisphere). Overall, 60 images per animal were analyzed. The software automatically converted all immunolabeled element beyond the threshold range into pure black pixels and converted the rest of the image into pure white pixels (Fig. 1). The software then calculated the percentage of pure black pixels for statistical analysis.

### Statistical analysis

All the data are presented as the mean ± standard error of the mean (S.E.M.). Differences among the groups were evaluated by using one-way ANOVA followed by the post hoc Fisher’s least significant different test. This analysis was performed with SPSS software (Dr. SPSS II for Windows v.11.0.1J, SPSS Japan Inc., Japan). A *p* value less than 0.05 was considered statistically significant.

## Results

### Effect of ECS on PPI deficits in Gunn rats

**Fig. 2:**
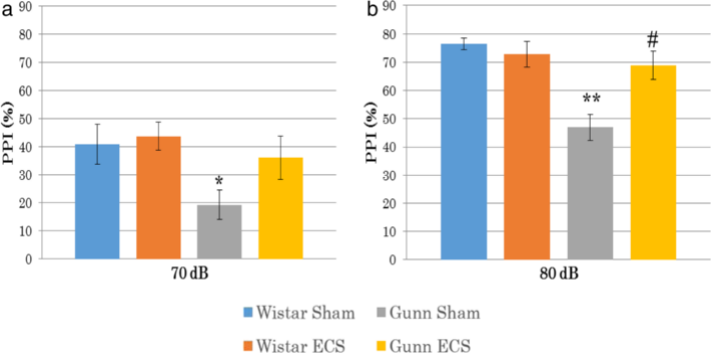
Effect of ECS on prepulse inhibition at 70 dB (**a**) and 80 dB (**b**). Each value is the mean ± S.E.M. (*n* = 8 per group). *p < 0.05, **p < 0.001 compared to the Wistar sham group. #*p* < 0.005, compared to the Gunn sham group

The PPI test was performed with two different prepulse stimulus intensities, namely 70 and 80 dB. At 70-dB prepulse stimulus intensity, %PPI was 40.94 ± 7.12 in the WS group, 43.81 ± 4.99 in the WE group, 19.32 ± 5.30 in the GS group, and 36.18 ± 7.72 in the GE group. As shown in Fig. 2a, %PPI at 70 dB was significantly decreased in the GS group compared to the WS group (*p* = 0.024). After consecutive administration of ECS for 6 days, we found a higher %PPI at 70 dB in the GE group compared to the GS group, although the ECS efficacy did not reach significance.

At 80-dB prepulse stimulus intensity, %PPI was 76.40 ± 2.17 in the WS group, 72.70 ± 4.45 in the WE group, 46.93 ± 4.67 in the GS group, and 68.87 ± 4.95 in the GE group. As shown in Fig. 2b, %PPI at 80 dB was significantly lower in the GS than the WS group (*p* < 0.001). After the ECS administration, at 80 dB, %PPI in the GE group was significantly increased compared to the GS group (*p* = 0.001), suggesting that ECS improved the schizophrenia-like behavior of Gunn rats. There was no significant difference between the WS group and the WE group both at 70 and at 80 dB.

**Fig. 3:**
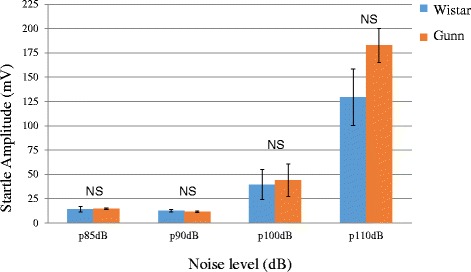
Startle amplitude of the Wistar group compared to the Gunn group at the noise level 85, 90, 100, and 110 dB. Each value is the mean ± S.E.M. (*n* = 3 per group). *NS* not significant

It is well known that both young [30] and adult [31] Gunn rats have changes in the brainstem auditory system. Shapiro and Hecox [31] have demonstrated that homozygous jaundiced Gunn rats have small but statistically significant abnormalities in the auditory system using brainstem auditory evoke potentials (BAEPs). On the other hand, Levi et al. [32] have shown that all homozygous jaundiced Gunn rats have preserved hearing ability in auditory nerve and brain stem responses, also known as BAEPs. Since studies of BAEPs in Gunn rats have found normal and abnormal auditory function, we determined whether Gunn rats have an intact hearing in our experimental system. We measured startle amplitude both in Wistar and Gunn rats (*n* = 3) by using the PPI test instrument at the noise level 85, 90, 100, and 110 dB (with 20-ms duration, white noise). As shown in Fig. 3, the startle response increased with increasing volume and reached a maximum at 110 dB both in Gunn and Wistar rats. At each noise level, there was no significant difference in startle response between Gunn and Wistar rats. Based on this result, we consider that Gunn rats show intact hearing in our experimental system using the PPI test instrument.

### Effect of ECS on microglial activation in Gunn rats

**Fig. 4:**
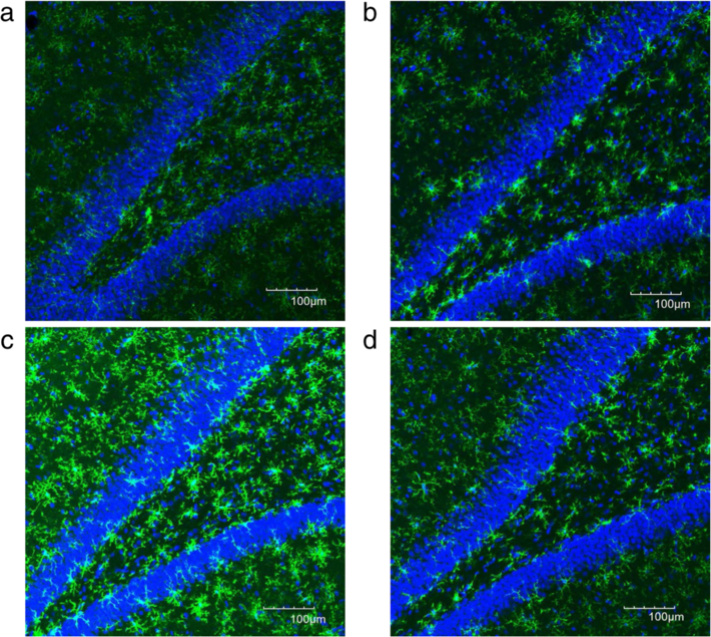
Representative immunofluorescent images of CD11b combined with DAPI in the DG area of the Wistar sham group (**a**), the Wistar ECS group (**b**), the Gunn sham group (**c**), and the Gunn ECS group (**d**). The scale bar indicates 100 μm

We evaluated the CD11b immunoreactivity in the DG, CA1, and CA3 regions of the hippocampus. In the DG, immunofluorescent images showed a high expression of CD11b in the GS group (Fig. 4c) compared to the WS group (Fig. 4a). The high expression of CD11b was considerably reduced, and the cell body of each microglia looks shrunk after ECS (Fig. 4d). Quantification of data for CD11b showed that CD11b immunoreactivity was significantly higher in the GS group compared to the WS group in the DG (*p* = 0.002) (Fig. 6a). After ECS, the CD11b immunoreactivity in the GE group was significantly decreased compared to the GS group (*p* = 0.038) (Fig. 6a).

**Fig. 5:**
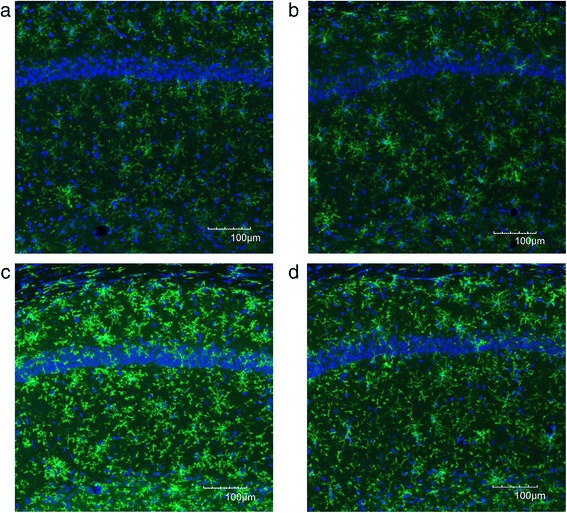
Representative immunofluorescent images of CD11b combined with DAPI in the CA1 of the Wistar sham group (**a**), the Wistar ECS group (**b**), the Gunn sham group (**c**), and the Gunn ECS group (**d**). The scale bar indicates 100 μm

**Fig. 6:**
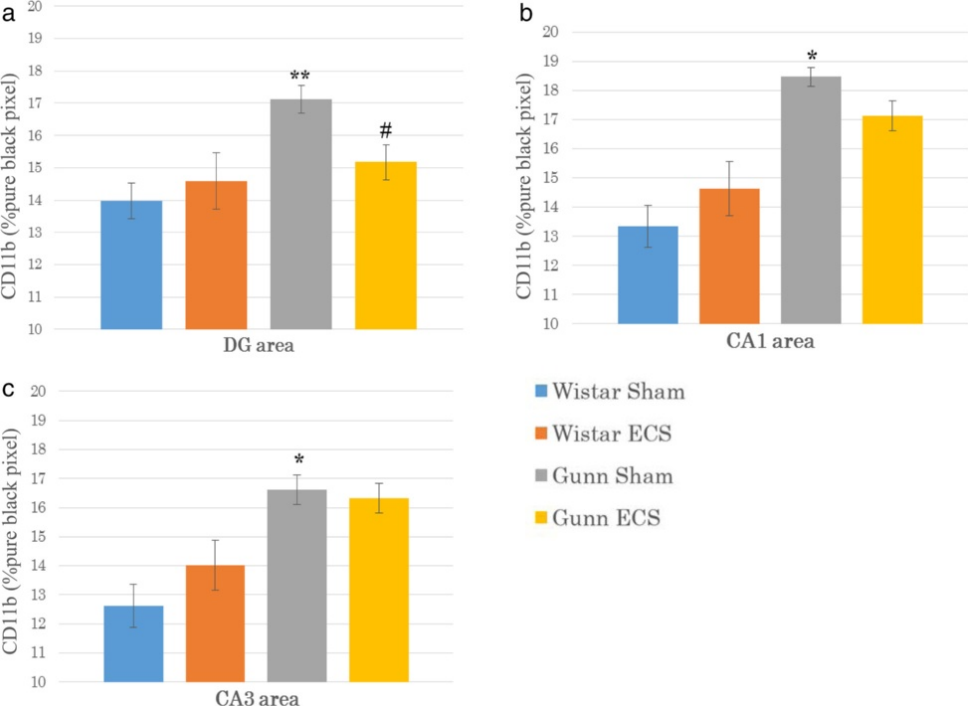
Effect of ECS on microglial activation. Mean percentage of pure black pixel indicating CD11b immunoreactivity in the DG area (**a**), the CA1 area (**b**), and the CA3 area (**c**). Each value is the mean ± S.E.M. (*n* = 6 per group). *p < 0.001, **p < 0.005, compared to the Wistar sham group. #*p* < 0.05, compared to the Gunn sham group. *DG* dentate gyrus, *CA* cornu ammonis

In the CA1, immunofluorescent images showed a high expression of CD11b in the GS group (Fig. 5c) compared to the WS group (Fig. 5a). ECS administration conferred the tendency to reduce the high expression of CD11b (Fig. 5d). Quantification of data for CD11b showed that CD11b immunoreactivity was also significantly higher in the GS group than the WS group in the CA1 (*p* < 0.001) (Fig. 6b) and in the CA3 (*p* < 0.001) (Fig. 6c). ECS administration showed a tendency to reduce the CD11b immunoreactivity, but it did not reach significance in these regions (Fig. 6b, c). No significant difference was observed between the WS group and the WE group in all three regions of the hippocampus.

### Effect of ECS on astrocytic activation in Gunn rats

**Fig. 7:**
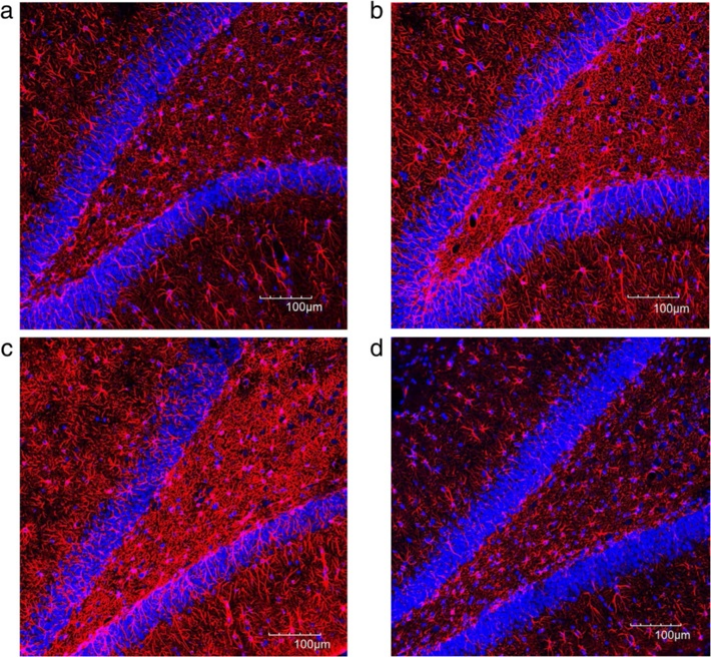
Representative immunofluorescent images of GFAP combined with DAPI in the DG of the Wistar sham group (**a**), the Wistar ECS group (**b**), the Gunn sham group (**c**), and the Gunn ECS group (**d**). The scale bar indicates 100 μm

We also examined the immunoreactivity of GFAP in the hippocampal DG, CA1, and CA3. Figure 7c represents immunofluorescent analysis and shows high expression of GFAP in the DG of the GS group compared to the WS group (Fig. 7a). ECS administration considerably decreased the GFAP immunoreactivity in the GE group (Fig. 7d). Quantitation of GFAP immunoreactivity in the DG showed that the GFAP expression in the GS group was significantly higher than in the WS group (*p* = 0.008) (Fig. 9a). The ECS administration significantly suppressed the increased GFAP immunoreactivity in the DG of Gunn rats (*p* = 0.004) (Fig. 9a).

**Fig. 8:**
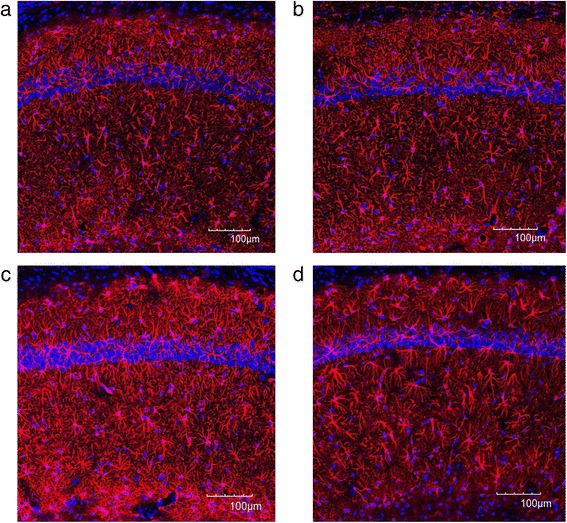
Representative immunofluorescent images of GFAP combined with DAPI in the CA1 of the Wistar sham group (**a**), the Wistar ECS group (**b**), the Gunn sham group (**c**), and the Gunn ECS group (**d**). The scale bar indicates 100 μm

**Fig. 9:**
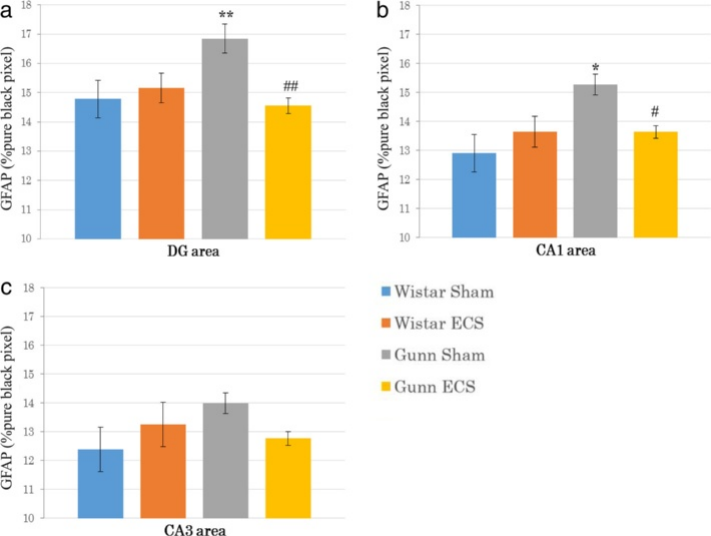
Effect of ECS on astrocytic activation. Mean percentage of pure black pixel indicating GFAP immunoreactivity in the DG area (**a**), the CA1 area (**b**), and the CA3 area (**c**). Each value is the mean ± S.E.M. (*n* = 6 per group). *p < 0.005, **p < 0.01, compared to the Wistar sham group. #*p* < 0.05, ##*p* < 0.005, compared to the Gunn sham group

Figure 8c is a representative immunofluorescent image which shows increased immunoreactivity of GFAP in the CA1 region of the GS group compared to the WS group (Fig. 8a). Such increased expression of GFAP was considerably reduced after ECS (Fig. 8d). Quantification of data for GFAP showed that the GFAP immunoreactivity in the CA1 was significantly higher in the GS group than in the WS group (*p* = 0.002) (Fig. 9b). The ECS administration significantly reduced the increased GFAP immunoreactivity in the CA1 of Gunn rats (*p* = 0.022) (Fig. 9b).

In the CA3 region, there was no significant difference between any two groups (Fig. 9c). There was no significant difference of the GFAP immunoreactivity between the WS group and the WE group in all the three regions.

## Discussion

There were three major findings in the present study. First, ECS administration significantly ameliorated the schizophrenia-like behavior in Gunn rats. Second, ECS inhibited microglial activation in the hippocampi of Gunn rats, as shown by the decreased immunoreactivity of CD11b. Third, ECS also attenuated astrocytic activation in the hippocampi of Gunn rats, as indicated by the reduced expression of GFAP. There have been several animal studies which evaluated the effects of ECS on glial cells in the normal brain [33–40]. However, to our knowledge, there have been only a few studies which examined the effects of ECS on glial cells in the pathological brain [41, 42]. The present study determined the effect of ECS on the microglial activation and astrocytic activation in the diseased brain by using Gunn rats.

Braff et al. [43–45] have shown that schizophrenic patients exhibited significant PPI deficits compared to normal participants. Moreover, numerous studies have confirmed the PPI deficiency in schizophrenic patients [46–48] and that antipsychotic drugs can not only ameliorate symptoms of schizophrenia but also improve the PPI deficit [49, 50]. Our previous studies have demonstrated that Gunn rats exhibit a schizophrenia-like behavior which consists of impaired sensorimotor gating as shown by decreased %PPI compared to Wistar rats, a normal rat strain [25, 26, 28]. Accordingly, Gunn rats showing such a schizophrenia-like behavior seems to be an appropriate animal model to investigate the therapeutic mechanism of ECS, the animal counterpart of ECT.

Continuous or maintenance ECT treatments have been reported to be effective as a relapse prevention treatment. Indications for maintenance ECT treatment include patients with rapid relapse after initial ECT, severe symptoms, psychotic symptoms, and the inability to tolerate medications [4, 51]. In a chart review by Kristensen, 18 patients received maintenance ECT in addition to antipsychotics. It was very effective in stabilizing the patients and reducing the length of hospital stay [52]. Another study has shown that continuous or maintenance ECT is safe and effective for chronically hospitalized patients. It improves general functioning and reduces verbal aggression and self-harm [53]. In the present study, consecutive administration of ECS for 6 days significantly ameliorated the impaired sensorimotor gating in Gunn rats as demonstrated by the increased percentage of PPI. Consistent with our finding, Chao et al. [54] have clarified that repeated ECS improves the PPI disruption caused by chronic administration of methamphetamine.

In schizophrenic patients, the disability to focus on what is important (i.e., attentional deficit) can be reflected by deficient attentional modulation of PPI [46, 55]. As attentional deficit is one of the core symptoms in attention-deficit/hyperactivity disorder (ADHD), the PPI test might be used as a test for ADHD. Schulz-Juergensen et al. [56] showed that the median baseline PPI of ADHD patients was below the value of age-matched normal controls and that methylphenidate significantly improved this deficiency. Although both schizophrenic patients and ADHD patients exhibit efficient attentional modulation of PPI, their attentional deficits are fundamentally different in some perspectives [57].

In this study, the hippocampus was intensively analyzed as the region of interest, since the hippocampus has been suggested to regulate PPI [58–60]. Furthermore, increasing evidence implies that the hippocampus is involved in the pathophysiology of schizophrenia [61–63] and is particularly vulnerable to inflammatory insults due to its high density of receptors for inflammatory mediators [64, 65].

The present study showed that CD11b expression was significantly increased in the hippocampal DG, CA1, and CA3 areas in Gunn rats compared to Wistar rats. The finding that microglia in the hippocampus of Gunn rats are activated is in line with our previous studies [28, 66]. Gunn rats have high levels of unconjugated bilirubin (UCB) in their blood [23]. Although UCB entrance into the brain is prevented by the blood-brain barrier (BBB), the free fraction of UCB still diffuses into the brain through the BBB [67–69] and causes glial activation [70, 71]. After ECS, we found a significant decrease in CD11b expression in the DG, but not in the CA1 and CA3. Our previous study using Gunn rats also showed that minocycline attenuated microglial activation in the hippocampal DG and thus improved the schizophrenia-like behavior [28]. Based on these findings, abnormal behavior similar to schizophrenia may be associated with microglial activation in the DG. Furthermore, ECS may inhibit microglial activation in the pathological brain, and this inhibitory effect on activated microglia may be a part of the therapeutic action of ECS.

Not only microglia, but astrocytes are also activated by UCB [72]. Astrocytes, like microglia, are activated in a response to injury or other pathological processes in the CNS and have either a neuroprotective or a neurotoxic role [73, 74]. In the present study, the level of GFAP expression in the hippocampi of Gunn rats was significantly increased compared to Wistar rats in the DG and CA1. After the ECS administrations, the GFAP expression was significantly decreased in the DG and CA1. The abnormal behavior in Gunn rats may be caused by high levels of UCB which may precede chronic inflammation and neurodegeneration in the Gunn rat brain. Therefore, it is presumed that activated astrocytes may play a neurotoxic role in Gunn rats and that ECS may exert therapeutic effect through inhibition of such activation of astrocytes.

Our previous study has indicated that Gunn rats show the increased number of apoptotic cells and reduced neurogenesis in the subgranular zone of their hippocampi [25]. Zarubenko et al. [75] have shown that repeated ECS causes neuronal death. On the other hand, a number of studies have shown that repeated ECS increases neurogenesis [76–78]. Moreover, Conti et al. [79] have demonstrated that nerve growth factor in the hippocampus was up-regulated after chronic ECS. Based on these findings, we presume that ECS may cause neuronal cell death, while ECS also induces neurogenesis to replace the death neuron. Not only neurogenesis, studies on the normal rodent brain have also shown that ECS increases angiogenesis [80, 81]. Accordingly, ECS may also induce neurogenesis and angiogenesis even in the pathological hippocampi of Gunn rats. The inhibitory effects of ECS on activated microglia and activated astrocytes seem to lead to the reduction of inflammatory activities in the pathological hippocampus. Thus, the ECS-induced generation of new neurons may replace the neurons damaged by the high levels of UCB, while the angiogenesis may repair the BBB and prevent the brain from excessive entrance of UCB. All these things induced by ECS appear to work together to improve the schizophrenia-like behavior in Gunn rats.

Our results showed that ECS significantly suppressed the CD11b expression only in the DG, not in the CA1 and CA3. A study on additional genes excitatory amino acid transporter-1 (EAAC1), that are known to be involved in neuroprotection, has shown that EAAC1 was significantly up-regulated in the DG following chronic ECS, while no changes were detected in other hippocampal subregions, including CA1 and CA3 [82]. It has been demonstrated that repeated ECS significantly increases the synaptic response in the DG only [83]. In addition, the significant inhibitory effect of ECS on the GFAP expression has been observed in the DG and CA1, but not in the CA3. Consistent with our finding, Conti et al. [79] have demonstrated that nerve growth factor in the hippocampus was up-regulated after chronic ECS in the DG and the CA1, but not in the CA3. Moreover, allopregnanolone infused into the CA1 of the hippocampus has been shown to enhance the PPI of startle response in Wistar rats [58]. Based on these findings, it is tempting to presume that the response to ECS treatment is regionally selective and the mechanism of ECS to improve PPI deficit may be related to the DG and CA1 rather than the CA3.

Mononuclear phagocytic cells may play a key role in the pathogenesis of major psychiatric disorders. In fact, a study by Rothermundt reported a slight increase in the mean absolute and relative monocyte counts of the schizophrenic patients [84]. Other studies also showed a monocytosis and a high number of CD14^+^ cells in untreated schizophrenia patients [85, 86]. Furthermore, in the cerebrospinal fluid of schizophrenic patients, there was an accumulation of monocytes and macrophages during acute psychotic episodes [87].

Our finding that ECS treatment inhibits activated glial cells in Gunn rats is inconsistent with an ECS study on normal rats by Jansson who demonstrated that ECS administration causes glial activation in several limbic regions, characterized by morphological changes and by the appearance of subpopulations of microglia and astrocytes [36]. Indeed, the majority of ECS studies on normal animals has shown that ECS has no effect on the activation/proliferation of microglia and astrocytes [34, 35] or even increases the glial activation/proliferation [38, 39]. Only one study has reported that ECS reduces the density of microglial process in the murine hippocampus [37] and one other has shown that ECS inhibits GFAP expression in the rat hippocampus [33]. Therefore, the effect of ECS on glial activation in the pathological brain may be different from that in the normal brain, and further studies on this issue are clearly warranted.

## Conclusions

In conclusion, our findings indicate that ECS on Gunn rats ameliorates schizophrenia-like behavior and attenuates microgliosis in the DG and astrogliosis in the DG and the CA1 of Gunn rats. Accordingly, therapeutic mechanism of ECT may be exerted in part by inhibition of glial activation. These results may also provide crucial information to elucidate the role of activated glia in the pathogenesis of schizophrenia and to determine whether future therapeutic interventions should attempt to up-regulate or down-regulate glial functions.

## Abbreviations

ECT: Electroconvulsive therapy
ECS: Electroconvulsive shock
TNF-α: Tumor necrosis factor-α
PPI: Prepulse inhibition
DG: Dentate gyrus
CA: Cornu ammonis
WS: Wistar sham group
WE: Wistar ECS group
GS: Gunn sham group
GE: Gunn ECS group
DAB: Diaminobenzidine
UCB: Unconjugated bilirubin
BBB: Blood-brain barrier
